# The molecular mechanisms of the long noncoding RNA SBF2-AS1 in regulating the proliferation of oesophageal squamous cell carcinoma

**DOI:** 10.1038/s41598-020-80817-w

**Published:** 2021-01-12

**Authors:** Wenjuan Zha, Xiaomin Li, Xiaowei Tie, Yao Xing, Hao Li, Fei Gao, Ting Ye, Wangqi Du, Rui Chen, Yangchen Liu

**Affiliations:** 1 Department of Radiotherapy, Taixing People’s Hospital Affiliated with Bengbu Medical College, Bengbu, China; 2 Department of Clinical Laboratory, Taixing People’s Hospital, Taixing, China; 3 Department of Radiotherapy, Taixing People’s Hospital, Taixing, 225400 China; 4 Department of Taixing People’s Hospital Affiliated with Yangzhou University, Yangzhou, 225000 China

**Keywords:** Cancer, Cell biology

## Abstract

The long noncoding RNASBF2-AS1 can promote the occurrence and development of many kinds of tumours, but its role in oesophageal squamous cell carcinoma (ESCC) is unknown. We found that SBF2-AS1 was up-regulated in ESCC, and its expression was positively correlated with tumor size (P = 0.0001), but was not related to gender, age, TNM stage, histological grade, and lymphnode metastasis (P > 0.05). It was further found that the higher the expression of SBF2-AS1, the lower the survival rate. COX multivariate analysis showed that the expression of SBF2-AS1 was an independent prognostic factor. Functional experiments show that inhibition of SBF2-AS1 can inhibit the proliferation of ESCC through in vivo and in vitro, and overexpression of SBF2-AS1 can promote the proliferation of ESCC and inhibit its apoptosis. In mechanism, SBF2-AS1/miR-338-3P, miR-362-3P/E2F1 axis are involved in the regulation of ESCC growth. In general, SBF2-AS1 may be used as ceRNA to combine with miR-338-3P and miR-362-3P to up-regulate the expression ofE2F1, and ultimately play a role in promoting cancer. It may be used as a therapeutic target and a biomarker for prognosis.

## Introduction

Oesophageal cancer is a common digestive tract tumour that ranks seventh in the world in terms of incidence and sixth in the world in terms of mortality, but oesophageal cancer ranks fifth in terms of incidence in China^1^. Oesophageal squamous cell carcinoma is one of the main subtypes of oesophageal cancer. Oesophageal squamous cell carcinoma (ESCC) is caused by the malignant transformation of normal oesophageal squamous epithelium, which usually occurs in the proximal oesophagus, caused by certain environmental factors, such as alcohol and smoking^2^. Although some studies have shown that the incidence of oesophageal squamous cell carcinoma is decreasing^3^, it remains very high, and the 5-year survival rate is less than 20%^4,5^. The occurrence and development of oesophageal squamous cell carcinoma are closely related to many genes. Long noncoding RNAs (lncRNAs) are a kind of RNA longer than 200 nt that does not encode a protein^6^. LncRNAs are involved in a wide range of biological processes. Almost every step in the gene life cycle, from transcription to mRNA splicing, RNA decay and translation, is affected by lncRNAs^7^. LncRNAs act mainly through four molecular mechanisms. First, lncRNAs can be used as a variety of molecular signals. Second, lncRNAs can function in molecular induction. Third, lncRNAs can be used as molecular guides to guide RNA-binding proteins. Fourth, lncRNAs can be used as a central platform for the assembly of related molecular components^8^. Therefore, imbalance between lncRNAs is an important causes of tumorigenesis. Many studies have shown that SBF2-AS1 can act as a cancer-promoting factor^9–11^. Zhang et al. found that SBF2-AS1 is positively correlated with the transcription factor ZEB1. The region of ZEB1 from − 684 to − 676 bp binds the SBF2-AS1 promoter and regulates SBF2-AS1 at the transcriptional level. Activated ZEB1 can promote the transcription of SBF2-AS1 to maintain the carcinogenicity of SBF2-AS1/miR-151a-3p/XRCC4^12^. Lu et al. found that SBF2-AS1 can bind EZH2 and especially SUZ12, the core components of PRC2, to thus bind PRC2. After the inhibition of SBF2-AS1, enrichment of the promoter region of P21 in SUV12 and EZH2 was significantly decreased, resulting in the upregulation of P21 expression. Overexpression of SBF2-AS1 reduced the expression of P21 and promoted tumorigenesis^13^. CeRNAs are responsible for a new kind of regulatory mechanism between noncoding RNAs and messenger RNAs^14^. An increasing number of studies have found that many lncRNAs mainly act as ceRNAs to regulate the occurrence and development of tumours, such as osteosarcoma^15^, non-small-cell lung cancer^16^, colorecta lcancer^17^ and glioma^18^. However, our mechanistic understanding of the network of ceRNAs that target SBF2-AS1 in oesophageal squamous cell carcinoma is not clear. In this study, SBF2-AS1 was used as a sponge to adsorb miR-338-3P and miR-362-3P, upregulating the expression of E2F1 and ultimately promoting the proliferation of oesophageal squamous cell carcinoma. Additionally, SBF2-AS1 could promote the proliferation of oesophageal squamous cell carcinoma in vivo and in vitro.

## Results

### The SBF2-AS1 is highly expressed in ESCC

**Figure 1:**
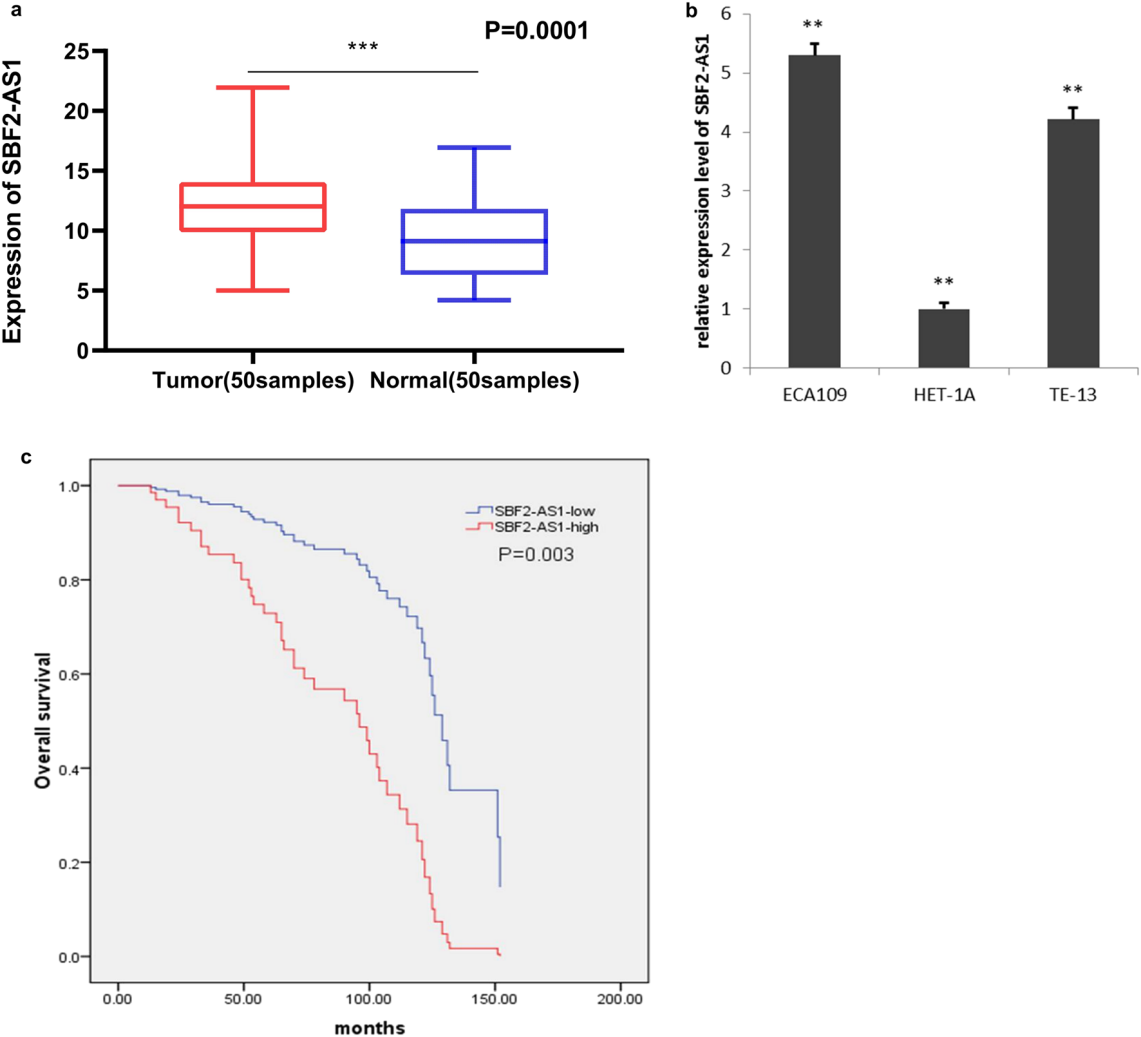
SBF2-AS1 is upregulated in ESCC tumours and cells, while miR-338-3P and miR-362-3P is low in ESCC. (**a**) Relative ratios between SBF2-AS1 expression in 50 ESCC tissues and that in50 normal oesophageal tissues. (**b**) The expression of SBF2-AS1 in oesophageal squamous cell carcinoma cells and normal oesophageal cells was detected by RT-PCR. (**c**) Analysis of overall survival in patients with low or high SBF2-AS1 expression levels. Data are shown as the mean ± SEM. *P < 0.05, **P < 0.01, ***P < 0.01.

SBF2-AS1 expression was examined in ESCC. The results showed that the expression level of SBF2-AS1 in ESCC tissues was higher than that in adjacent normal tissues (Fig. 1a). Additionally, SBF2-AS1 was found to be up regulated in ESCC cell lines (Fig. 1b). These results suggest that SBF2-AS1 is highly expressed in ESCC tissues and cells.

### High levels of SBF2-AS1 expression correlate with clinicopathologic features and poor survival in ESCC

In order to study the relationship between the expression level of SBF2-AS1 and the clinical pathological characteristics of patients, patients were divided into SBF2-AS1 high expression group and SBF2-AS1 low expression group. The results showed that the expression level of SBF2-AS1 was positively correlated with tumor size (P = 0.0001). The higher the expression level of SBF2-AS1, the larger the tumor, but there were no statistical differences in gender, age, TNM stage, histological grade, and lymph node metastasis (Table 1). And further research found that the expression level of SBF2-AS1 is negatively correlated with the overall survival time of the patient. The higher the expression level of SBF2-AS1, the shorter the overall survival time of the patient (Fig. 1c). COX multivariate analysis further shows that the expression level of SBF2-AS1 is an independent factor predicting the poor prognosis of patients with ESCC (Table 2).

**Table 1:** The correlation between clinicopathological parameters and SBF2-AS1 expression in human esophageal cancer.

Clinical features	Total (N = 100)	SBF2-AS1		p-value
		High = 50	Low = 50	
**Age (years)**				0.0650
≤ 65	39	15	24	
> 65	61	35	26	
**Gender**				0.1422
Male	65	29	36	
Female	35	21	14	
**TNM stage**				0.9999
pT1-pT2	46	23	23	
PT3-pT4	54	27	27	
**Histological grade**				0.8407
Low	45	23	22	
High	55	27	28	
**Tumor size (cm)**				0.0001
≤ 3	48	11	37	
> 3	52	39	13	
**Lymph node metastasis**				0.6882
No	46	22	24	
Yes	54	28	26	

Pearson chi-square test was used for comparison between subgroups. Bold values indicate p-value < 0.05.

**Table 2:** Multivariate analysis for overall survival by cox regression model.

Factors	Univariate cox			Multivariate cox		
	Exp(B)	95 CI%	Sig	Exp(B)	95 CI%	Sig
Age	1.659	0.863–3.227	0.136			NS
Gender	1.368	0.699–2.678	0.361			NS
TNM stage	1.009	0.549–1.856	0.977			NS
Lymphnod metastasis	1.112	0.611–2.074	0.727			NS
Histological grade	0.840	0.463–1.525	0.568			NS
Tumor size	2.023	1.075–3.809	0.029			NS
SBF2-AS1	5.006	2.324–10.781	0.0001	3.900	1.613–9.431	0.003

### Overexpression of SBF2-AS1 promoted ESCC cell proliferation

**Figure 2:**
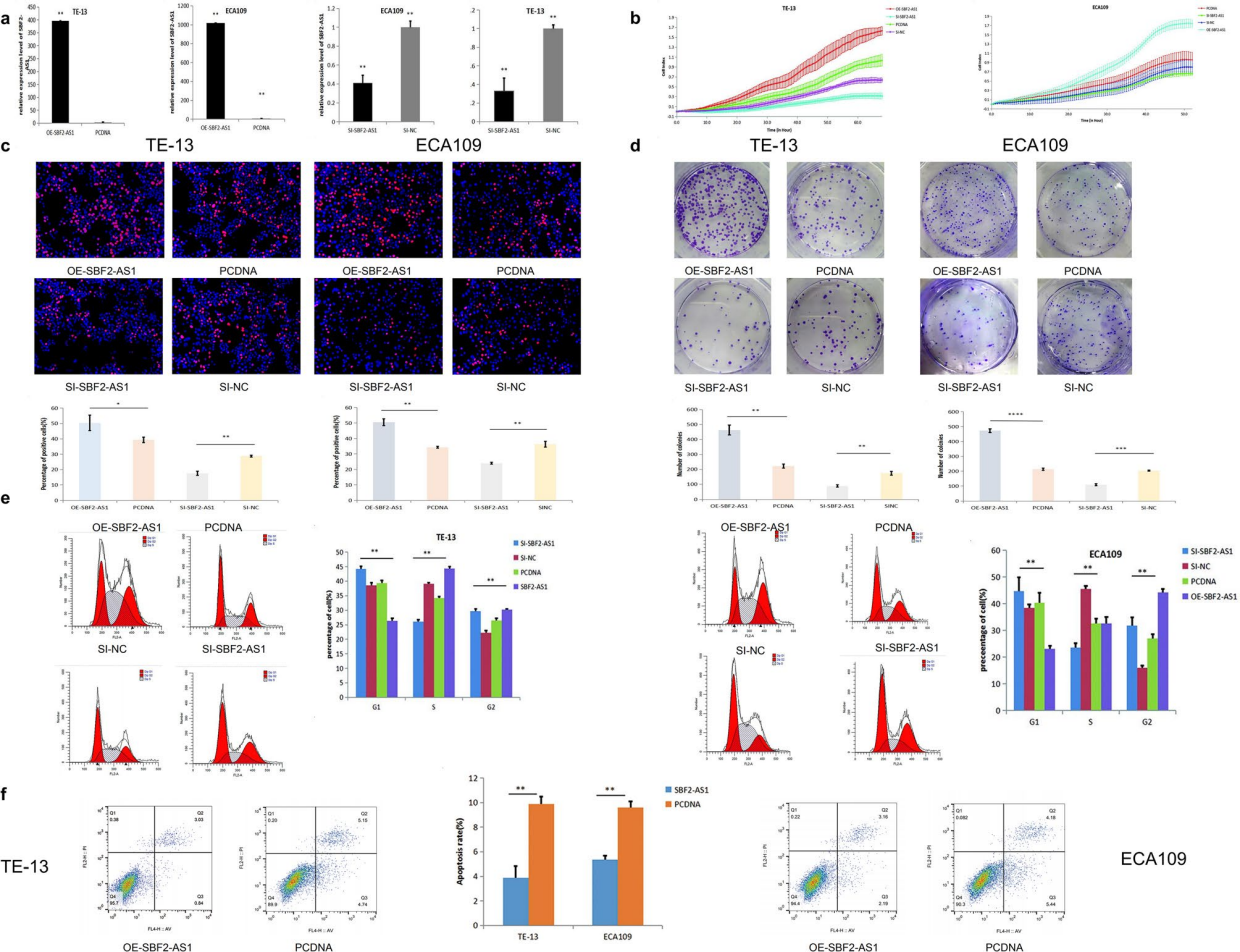
SBF2-AS1 promotes the proliferation of oesophageal squamous cell carcinoma cell lines (ECA109 and TE-13 cells). (**a**) SBF2-AS1 overexpression plasmid, a negative control, SI-SBF2-AS1 and SI-NC were transiently transfected into ECA109 and TE-13 cells, and the expression of SBF2-AS1 was analysed by RT-PCR. (**b**) Analysis of cell proliferation. The SBF2-AS1 overexpression plasmid, a negative control, SI-SBF2-AS1 and SI-NC were transiently transfected into ECA109 and TE-13 cells, and cell proliferation was detected by RTCA. (**c**) Analysis of cell proliferation. The SBF2-AS1 overexpression plasmid, a negative control, SI-SBF2-AS1 and SI-NC were transiently transfected into ECA109 and TE-13 cells, and cell proliferation was detected by EdU assay. (**d**) Colony formation assay. The SBF2-AS1 overexpression plasmid, a negative control, SI-SBF2-AS1 and SI-NC were transiently transfected into ECA109 and TE-13 cells, and colony formation was analysed by colony formation assay. (**e**) Cell cycle analysis. The SBF2-AS1 overexpression plasmid, a negative control, SI-SBF2-AS1 and SI-NC were transiently transfected into ECA109 and TE-13 cells, and the proportion of cells at each stage of the cell cycle was analysed. (**f**) Apoptosis analysis. The SBF2-AS1 overexpression plasmid and negative control were transiently transfected into ECA109 and TE-13 cells, the apoptosis rate of the cells was analysed. Data are shown as the mean ± SEM. *P < 0.05, **P < 0.01, ***P < 0.01.

To analyse the effect of SBF2-AS1 on oesophageal squamous cell carcinoma cell lines, ECA109 and TE-13 cells were transfected with OE-SBF2-AS1, PCDNA, si-SBF2-AS1 and si-NC. RT-PCR showed that the expression of SBF2-AS1 in the overexpression group was significantly higher than that in the control group, while that in the si-SBF2-AS1 group was significantly lower than that in the control group (Fig. 2a). The RTCA results showed that SBF2-AS1 significantly promoted the proliferation of oesophageal squamous cell carcinoma, while si-SBF2-AS1 inhibited the proliferation of oesophageal squamous cell carcinoma (Fig. 2b). EdU and colony formation assays further showed that SBF2-AS1 could promote the proliferation of oesophageal squamous cell carcinoma (Fig. 2c,d). Flow cytometry analysis showed that SBF2-AS1 could promote the transition from G1 phase to S phase, and flow cytometry to analyse cell apoptosis showed that SBF2-AS1 could inhibit the apoptosis of oesophageal cancer cells (Fig. 2e,f).

### SBF2-AS1 can adsorb miR-362-3P and miR-338-3P as a ceRNA

**Figure 3:**
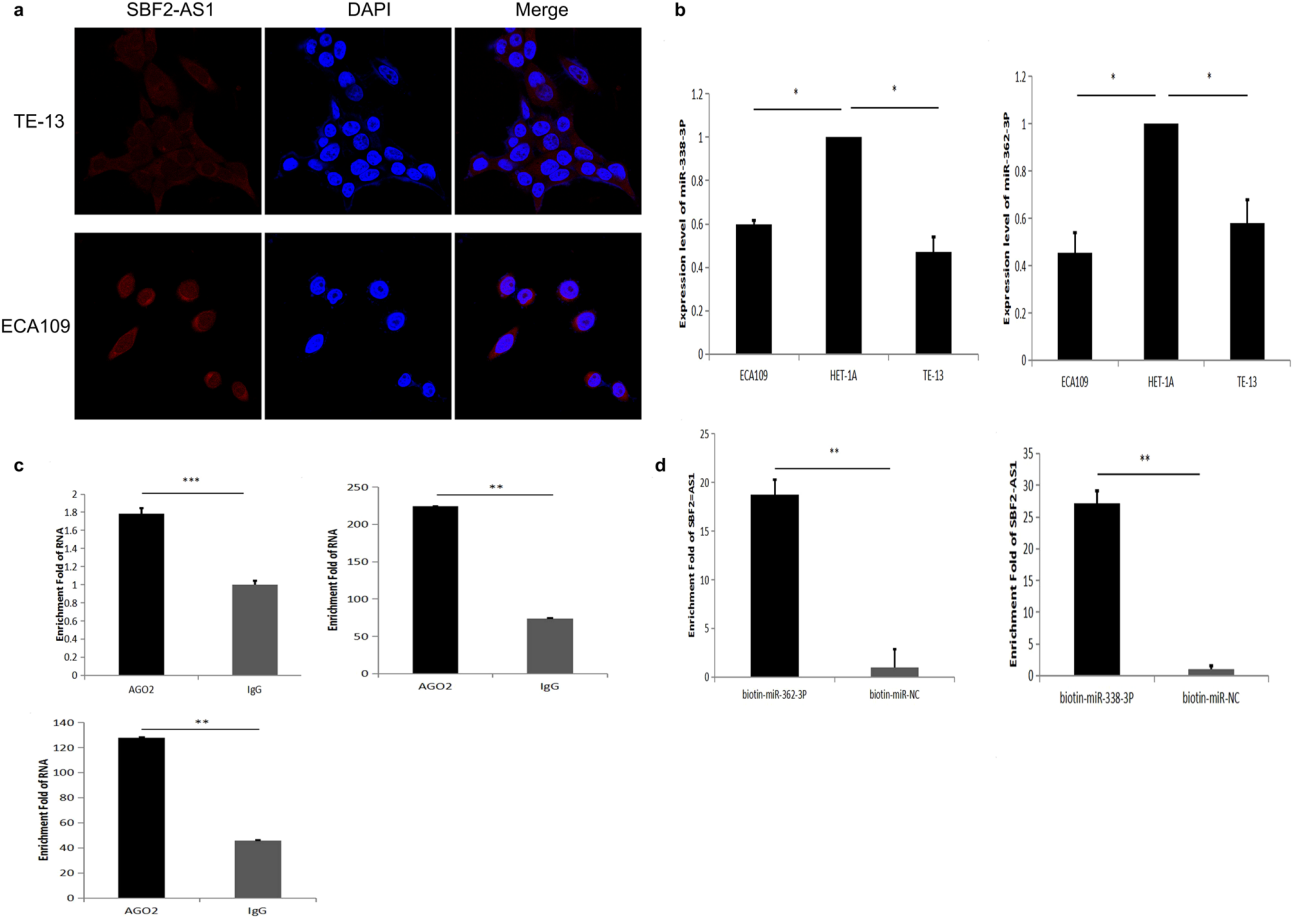
SBF2-AS1 is a sponge RNA for miR-338-3P and miR-362-3P. (**a**) Localization of SBF2-AS1 in oesophageal squamous cell carcinoma. (**b**) The expression of miR-338-3P and miR-362-3P in oesophageal squamous cell carcinoma cells and normal oesophageal cells was detected by RT-PCR. (**c**) A RIP assay using an antibody specifically targeting Ago2 showed that SBF2-AS1 could bind the Ago2 protein in ECA109 cells. (**d**) RNA-pull down assay. Biotin-miR-338-3P, biotin-miR-362-3P and biotin-miR-NC were transiently transfected into ECA109 cells, and enrichment in SBF2-AS1 was determined and compared. Data are shown as the mean ± SEM. *P < 0.05, **P < 0.01, ***P < 0.01.

To determine the molecular mechanism by which SBF2-AS1 regulates ESCC, we first confirmed that SBF2-AS1 is mainly located in the cytoplasm through FISH experiments (Fig. 3a). Then, the downstream target of SBF2-AS1 was predicted by using the starBase website (http://starbase.sysu.edu.cn/), and SBF2-AS1 was found to bind miR-338-3P and miR-362-3P. And we also detected the expression levels of miR-338-3P and miR-362-3P in ESCC lines, and found that miR-338-3P andmiR-362-3P were low expressed in ESCC lines (Fig. 3b). Many studies have reported that SBF2-AS1 can be used as a ceRNA to promote the occurrence and development of tumours. And whether it can bind to AGO2 protein is regarded as an important marker to play the role of ceRNA. To confirm that SBF2-AS1 can be used as a ceRNA to adsorb miR-338-3P and miR-362-3P, we used RNA immunoprecipitation (RIP) to detect whether SBF2-AS1 could bind the AGO2 protein in oesophageal cancer cells. The results showed that the AGO2 protein bound more SBF2-AS1 than IgG, suggesting that SBF2-AS1 can act as a ceRNA (Fig. 3c). Furthermore, we carried out RNA-pull down experiments with biotin-labelled miR-338-3P and miR-362-3P in ECA109 cells and found that biotin-labelled miR-338-3P and miR-362-3P could bind more SBF2-AS1 than that bound in the control group (Fig. 3d). Therefore, it was confirmed that SBF2-AS1 can be used as a ceRNA to adsorb miR-338-3P and miR-362-3P.

### MiR-338-3P and miR-362-3P can bind E2F1

**Figure 4:**
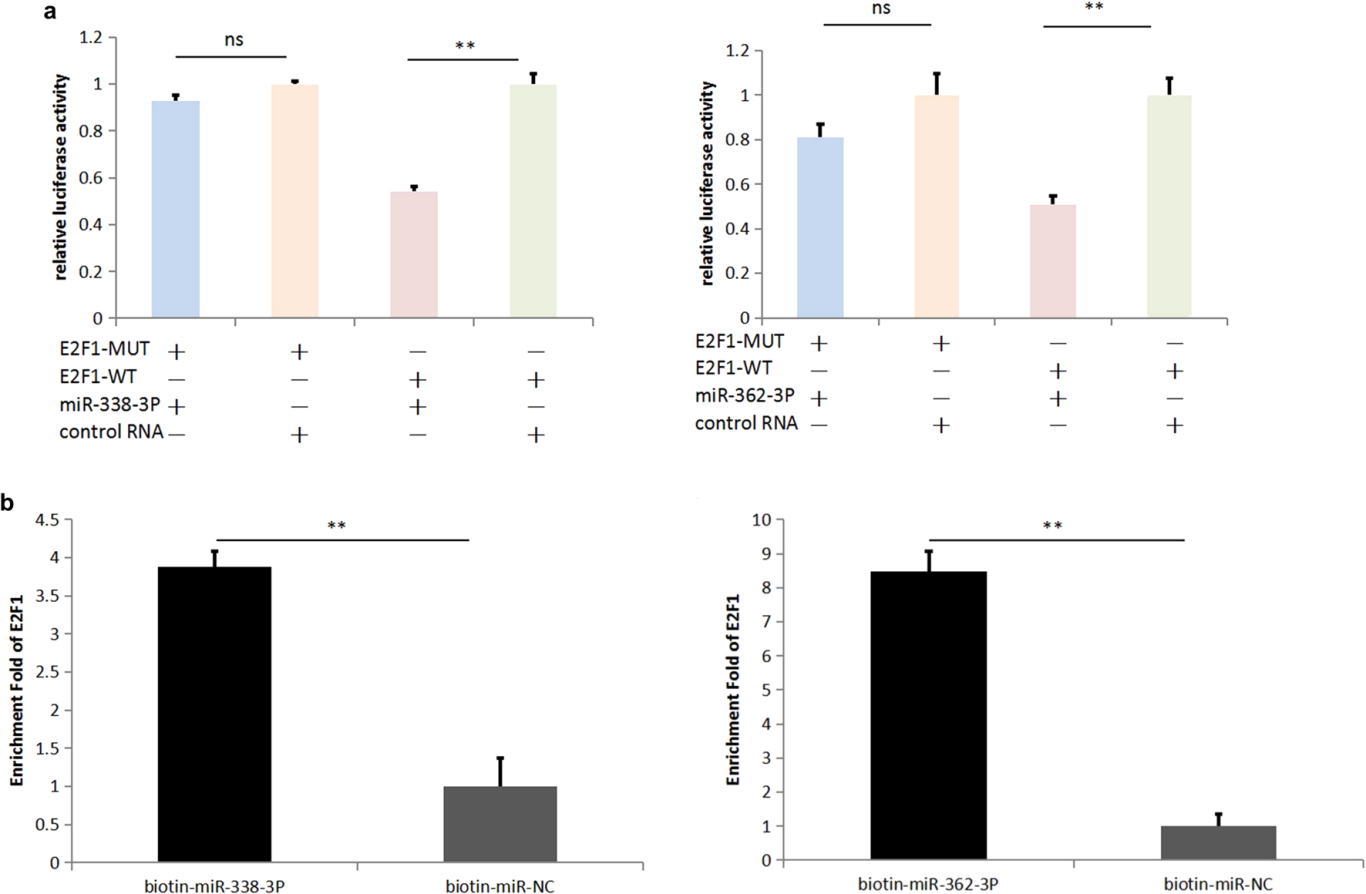
MiR-338-3P and miR-362-3P can bind E2F1. (**a**) Dual-luciferase reporter assays. pmirGLO-E2F1 WT, pmirGLO-E2F1 MUT1 (in which the binding site for miR-338-3p was mutated), and pmirGLO-E2F1 MUT2 (in which the binding site for miR-362-3p was mutated) were cotransfected into ECA109 cells with miR-338-3p and miR-338-3p, respectively, after which the cells were subjected to dual-luciferase assays. (**b**) RNA-pull down assay. Biotin-miR-338-3P, biotin-miR-362-3P and biotin-miR-NC were transiently transfected into ECA109 cells, and their enrichment in SBF2-AS1 was determined and compared. Data are shown as the mean ± SEM. *P < 0.05, **P < 0.01, ***P < 0.01, *N.S.* no statistical significance.

To identify the downstream target of miR-338-3P and miR-362-3P, we used the TargetScan website (https://www.targetscan.org/vert_72/) to predict the RNA downstream of miR-338-3P and miR-362-3P and found the presence of binding sites between E2F1 and miR-362-3P/miR338-3P. To confirm that miR-338-3P and miR-362-3P can bind E2F1, we designed wild-type and mutant E2F1 plasmids. The binding of miR-338-3P and miR-362-3P to E2F1 was confirmed by dual-luciferase reporter gene assays (Fig. 4a). RNA-pulldown experiments were carried out with biotin-labelled miR-338-3P and miR-362-3P, which revealed that miR-338-3P and miR-362-3P bind more E2F1 than that bound in the control group (Fig. 4b). These findings suggest that E2F1 is a downstream target of miR-338-3P and miR-362-3P.

### SBF2-AS1 can upregulate the expression of E2F1 by adsorbing miR-338-3P and miR-362-3P

**Figure 5:**
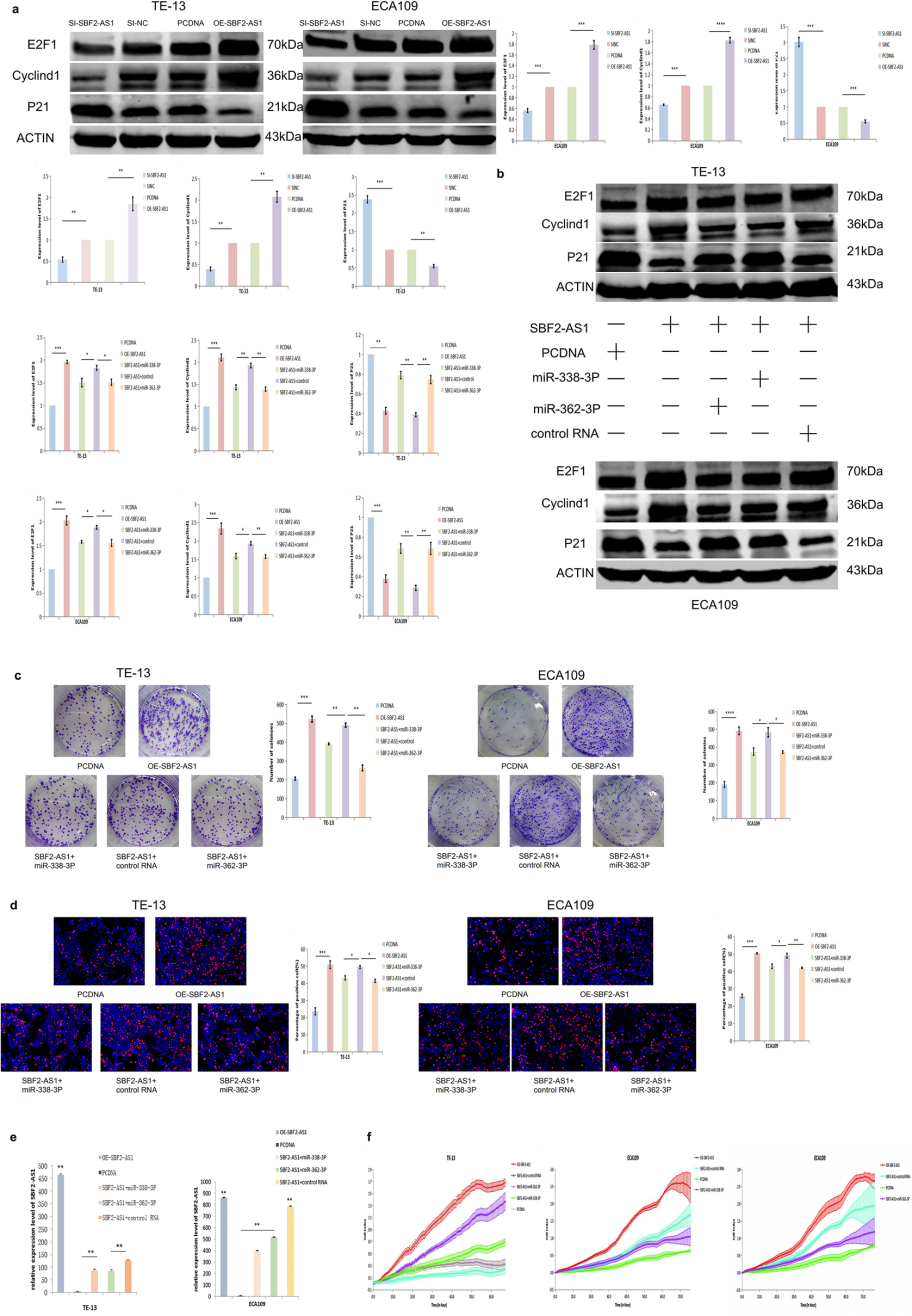
SBF2-AS1 in combination with miR-338-3P and miR-362-3P can increase the expression of E2F1 to promote the proliferation of oesophageal squamous cell carcinoma. (**a**) Western blot assay (samples derived from another experiment and the blots were processed in parallel). The SBF2-AS1 overexpression plasmid, a negative control, SI-SBF2-AS1 and SI-NC were transiently transfected into ECA109 and TE-13 cells, which were subjected to Western blot analysis of E2F1, Cyclind1 and P21 protein expression. (**b**) Western blot assay (samples derived for another experiment and the blots were processed in parallel). The SBF2-AS1 overexpression plasmid, a negative control, the SBF2-AS1 overexpression plasmid + miR-338-3P, and the SBF2-AS1 overexpression plasmid + miR-362-3P were transiently transfected into ECA109 and TE-13 cells, which were subjected to Western blot analysis of E2F1, Cyclind1 and P21 protein expression. (**c**) Colony formation assay. The SBF2-AS1 overexpression plasmid, a negative control, the SBF2-AS1 overexpression plasmid + miR-338-3P, and the SBF2-AS1 overexpression plasmid + miR-362-3P were transiently transfected into ECA109 and TE-13 cells, and colony formation ability was analysed by colony formation assay. (**d**) Analysis of cell proliferation. The SBF2-AS1 overexpression plasmid, a negative control, the SBF2-AS1 overexpression plasmid + miR-338-3P, and the SBF2-AS1 overexpression plasmid + miR-362-3P were transiently transfected into ECA109 and TE-13 cells, and cell proliferation was detected by EdU assay. (**e**) Analysis of cell proliferation. The SBF2-AS1 overexpression plasmid, a negative control, the SBF2-AS1 overexpression plasmid + miR-338-3P, and the SBF2-AS1 overexpression plasmid + miR-362-3P were transiently transfected into ECA109 and TE-13 cells, and the expression of SBF2-AS1 was analysed by RT-PCR. (**f**) Analysis of cell proliferation. The SBF2-AS1 overexpression plasmid, a negative control, the SBF2-AS1 overexpression plasmid + miR-338-3P, and the SBF2-AS1 overexpression plasmid + miR-362-3P were transiently transfected into ECA109 and TE-13 cells, and cell proliferation was detected by RTCA. Data are shown as the mean ± SEM. *P < 0.05, **P < 0.01, ***P < 0.01.

To show that SBF2-AS1 upregulates the expression of E2F1 by adsorbing miR-338-3P and miR-362-3P, we designed a series of cross-rescue experiments. Expression of the E2F1 protein in two oesophageal cancer cell lines, ECA109 andTE-13 cells, was detected by Western blot assay. The results showed that the overexpression of SBF2-AS1 promoted the expression of E2F1, while si-SBF2-AS1 inhibited the expression of E2F1. At the same time, the expression levels of Cyclind1 andP21, which are closely related to the cell cycle, was detected. The results showed that SBF2-AS1promoted the expression of Cyclind1 and inhibited the expression of P21 (Fig. 5a). Western blot analysis also proved that miR-338-3P and miR-362-3P could abrogate the upregulated expression of E2F1 and Cyclind1 and downregulated P21 expression caused by SBF2-AS1 (Fig. 5b). Colony-formation, EdU and RTCA rescue assays showed that miR-338-3P and miR-362 could attenuate the effect of SBF2-AS1 on cell proliferation (Fig. 5c,d,f). QRT-PCR rescue experiment showed that miR-338-3P and miR-362-3P decreased the expression level of SBF2-AS1 (Fig. 5e). These findings suggest that SBF2-AS1, acting as a ceRNA, adsorbs miR-338-3P and miR-362-3P and upregulates E2F1 to promote the proliferation of oesophageal cancer cells.

### SBF2-AS1ASO could inhibit the proliferation of oesophageal cancer cells in vivo

**Figure 6:**
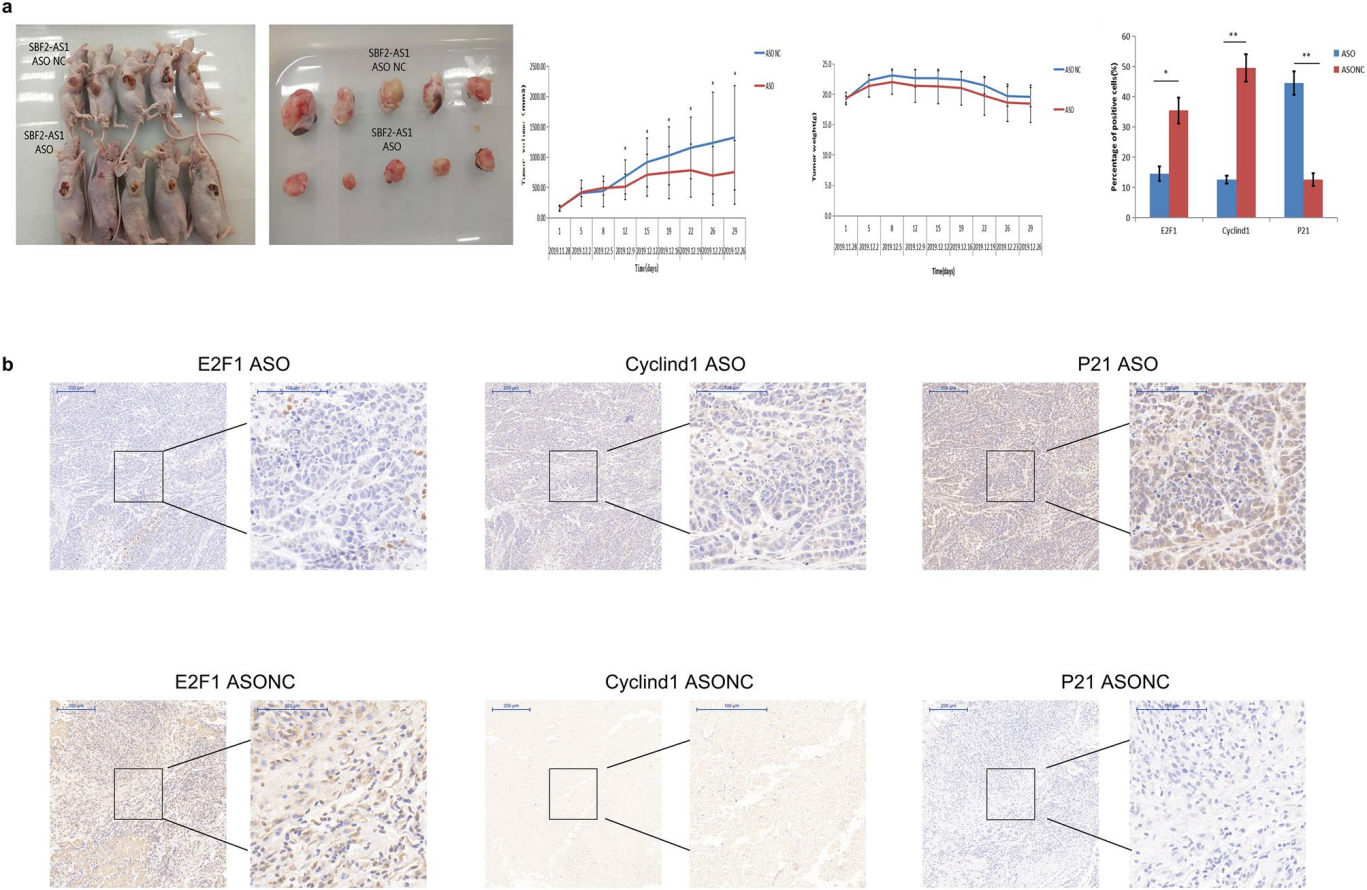
SBF2-AS1ASO could inhibit the growth of oesophageal squamous cell carcinoma in vivo. (**a**) Nude mouse xenograft model. Changes in mouse bodyweight and tumour volume in the nude mouse xenograft model and photographs of tumour xenografts are shown. (**b**) IHC staining of xenograft tumour tissues from mice in the SBF2-AS1 ASO group and SBF2-AS1 ASONC group is shown. Data are shown as the mean ± SEM. *P < 0.05, *N.S.* not statistically significant.

To investigate whether SBF2-AS1 interference could inhibit the proliferation of ESCC in vivo, we designed SBF2-AS1 ASO, a siRNA with a therapeutic effect. We used male SPF-grade Balb/C nude mice to carry out a subcutaneous tumour-bearing experiment. The mice were divided into the ASONC group and ASO group. The results showed that the tumour volume in the ASO group was significantly smaller than that in the ASONC group (Fig. 6a). Staining for E2F1 by immunohistochemistry was further analysed in these two groups, and the staining intensity of E2F1 in the ASO group was found to be significantly lower than that in the ASONC group (Fig. 6b). Therefore, SBF2-AS1 ASO could inhibit the growth of oesophageal cancer in vivo.

## Discussion

Eukaryotic cells can transcribe many types of RNA, including protein-encoding RNAs, short non-coding RNAs and long non-coding RNAs^19^. According to their location in the genome, lncRNAs are divided into five types: sense lncRNAs, antisense lncRNAs, bidirectional lncRNAs, intra-gene lncRNAs and genomic lncRNAs^20^. These RNAs are involved in many disease processes^21^ and participate in the occurrence and development of many kinds of malignant tumours and drug resistance in many malignant tumour types. LncRNAs regulate the occurrence and development of many kinds of malignant tumours, such as rectal cancer^22^, gastric cancer^23^, lung cancer^24^, cervical cancer^25^ and prostate cancer^26^.The lncRNA SBF2-AS1 is also involved in the formation of a variety of tumours^27–29^. Many lncRNAs have been demonstrated to be related to the occurrence of oesophageal squamous cell carcinoma, and many lncRNAs can cause a poor prognosis in patients with oesophageal squamous cell carcinoma^30^. In this study, we found that SBF2-AS1 was highly expressed in a variety of tumours and then analysed the TCGA database, which revealed that the expression level of SBF2-AS1 was significantly higher in oesophageal squamous cell carcinoma tissues than in normal tissues. Previous studies have shown that SBF2-AS1 can promote the development of gastric cancer through sponging miR-545^31^. Next, we wanted to determine whether SBF2-AS1 sponges other miRNAs to regulate the development of oesophageal squamous cell carcinoma. In our study, we found networks of ceRNAs against SBF2-AS1, miR-338-3P and E2F1 in EC109 cells. Through bioinformatics analysis, we found that miR-338-3P and miR-362-3P may be downstream target genes of SBF2-AS1. The tumour suppressor genes miR-338-3P-32^32,33^ and miR-362-3P^34,35^ are downregulated in many tumours such as ESCC. In this study, RIP and RNA-pull down experiments showed that SBF2-AS1 could directly target miR-338-3P and miR-362-3P, which confirmed the results of bioinformatics analysis. In terms of an effect on cell proliferation, the overexpression of SBF2-AS1 alone could promote cell proliferation, while co-transfection with miR-338-3P and miR-362-3P abrogated the proliferation of tumour cells promoted by SBF2-AS1. These results indicate that SBF2-AS1 plays the role of a ceRNA against miR-338-3P and miR-362-3P. An increasing number of studies have shown that lncRNAs, miRNAs, and mRNAs form the core of the ceRNA network^36^. Through use of the TargetScan database, we identified a common target gene of miR-338-3P and miR-362-3P: EF21. Previous studies have shown that E2F1 is an oncogene that can promote the occurrence and development of malignant tumours, such as breastcancer^37^. Some studies have shown that lncRNAs are involved in regulation of the miRNA/E2F1 axis in malignant tumours. For example, the lncRNA FER1L4 can downregulate the expression of miR-372 and enhance the expression of E2F1 in gliomas^38^. The lncRNA NNT-AS1 can promote the carcinogenesis and cell cycle progression of gastric cancer through the miR-424/E2F1 axis^39^. The results of this study showed that SBF2-AS1 enhanced expression of the E2F1 protein through acting as a sponge for miR-338-3P and miR-362-3P in ECA109 and TE-13 cells. The rescue experiment further showed that SBF2-AS1 promoted the growth of oesophageal squamous cell carcinoma cells through the miR-338-3P-miR-362-3P/E2F1 axis. Our study shows that the SBF2-AS1/miR-338-3P-miR362-3P/E2F1 axis is a potential mechanism for the malignant growth of oesophageal squamous cell carcinoma.

Overall, our study confirmed the high expression of SBF2-AS1 in oesophageal squamous cell carcinoma. SBF2-AS1 promotes the occurrence and development of oesophageal squamous cell carcinoma through sponging miR-338-3P andmiR-362-3P, which regulates the expression of E2F1. This study suggests that SBF2-AS1 may be a molecular marker of the malignant proliferation of oesophageal squamous cell carcinoma.

## Materials and methods

### Tissue specimens

Specimens were collected from 150 patients at Taixing People's Hospital, and all specimens were frozen at -80 °C. With regard to the collection of specimens, all the patients signed an informed consent form, approval was obtained from the Ethics Committee of Taixing People's Hospital, and the experiment was carried out in accordance with the regulations of the Ethics Committee of Bengbu Medical College.

### Transfection and treatment

ECA109 and TE-13 cells were obtained from the laboratory of Taixing People’s Hospital. The ECA109 and TE-13 cells were cultured in RPMI 1640 medium (GIBCO-BRL, Invitrogen, Carlsbad, CA, USA) containing 10% foetal bovine serum (FBS) and cultured in an in cubator with 5% CO_2_ at 37 °C. When the cells had grown to 90–100% confluence, they were passaged at a 1:2 ratio. The SBF2-AS1 overexpression plasmid, negative control (PCDNA), SI-SBF2-AS1 and SI-NC was constructed by Ruizhen Biotechnology Co., Ltd (Nanjing, China). The cells (10^6^) were plated on a six-well plate. When the cells grew to 70–80% confluence, Lipofectamine 3000 (Invitrogen, USA, L3000015) was used to transfect the SBF2-AS1 overexpression plasmid (OE-SBF2-AS1) and PCDNA, and RNAiMAX (Invitrogen, USA, 13778150) was used for SI-NC and SI-SBF2-AS1 transfection.

### RNA extraction and qPCR

RNA was extracted with TRIzol according to the manufacturer's instructions (Life Technologies, Scotland, UK). The extracted RNA was reverse transcribed into cDNA using PrimeScript RT Master Mix (Takara, Catalogue No. RR036A). DNAMAN software was used to design primers according to the principle of primer design. qPCR was performed on a Bio-Rad CFX-96 system (Bio-Rad). The primers used are listed in the attached file.

### Fluorescence in situ hybridization (FISH)

Through previous experiments, SBF2-AS1 was found to be mainly located in the cytoplasm, and detection of the subcellular localization of SBF2-AS1 was carried out with a Ribo Fluorescent in situ hybridization kit (RiboBio, Guangzhou, China, R11060.6). The cells were plated on a 24-wellplate, washed with PBS for 5 min after 24 h, and fixed with 4% paraformaldehyde for 10 min at room temperature. After washing with PBS three times, the cells were incubated with permeabilization buffer for 5 min at 4 °C. The cells were then sealed with pre-hybrid solution for 30 min at 37 °C, after which 2.5 µl of the SBF2-AS1 probe designed by Ribo was added to hybrid solution preheated to 37 °C. The pre-hybrid solution was discarded, and the hybrid solution containing probe was added and incubated overnight at 37 °C. Then, the cells were sequentially washed with hybrid solutions I, II and II and then stained with DAPI staining solution for 10 min, after which the cells were washed with PBS three times for fluorescence detection. Finally, a ZEISS microscope was used to acquire images under 40 × magnification.

### RTCA

Six-well plates were seeded with cells after they had grown to 80%-90% confluence. Twenty-four hours later, cell transfection was carried out. One day later, the baseline was measured by the addition of 50 µl of complete medium to each well of the E-plate. A total of 5 × 10^3^ cells from each group were added to wells in the E-plate, and the data were then measured with an xCELLigence Real Time Cell Analysis System (ACEA Biosciences, 380601050).

### Clone formation experiment

A total of 400 cells in RPMI 1640 medium were added to each well of a six-well plate and then washed twice with pre-cooled PBS. The cells were then fixed with 4% paraformaldehyde at room temperature for 15 min and washed twice with PBS. Then, the cells were incubated for 20 min at room temperature with 1% crystal violet. Finally, tap water was used to slowly flush away the excess crystal violet.

### 5-Ethynyl-2′-deoxyuridine

The EdU assay was carried out with the Cell-Light EdU Apollo 567 In Vitro Kit (RiboBio, Guangzhou, China, C10310-1). Cells in logarithmic growth phase were inoculated in a 96-well plate with 4 × 10^3^–1 × 10^4^ cells per well, and the cells were transfected 24 h later. Twenty-four hours after transfection, EdU solution was added to the cells and incubated for 2 h, after which the cells were washed twice with PBS for 5 min each. A cell fixation solution (50 µl) was added and incubated for 30 min at room temperature, after which the fixation solution was discarded. Fifty microliters of glycine at 2 mg/ml was added to each well and incubated for 5 min to stain the cells. Then, the cells were washed for 5 min with pre-cooled PBS. Then, 100 µl of penetrant was added to each well for incubation for 10 min and washed for 5 min with PBS. Then, 100 µl of a 1 × Apollo dye solution was added to each well and incubated for 30 min to stain the cells, after which 100 µl of penetrant was added to each well to wash the cells for 10 min. Finally, the cells were incubated in a shaker with a 1 × Hoechst 33342 solution for 30 min and then washed with PBS twice for fluorescence detection. Finally, images were acquired under 20 × magnification with a light microscope.

### Cell cycle analysis

Cells in a six-well plate were fixed with 500 µl of 75% ethanol at 4 °C for 4 h. Then, the cells were centrifuged at 1500 rpm for 5 min and washed once with PBS, and 400 µl of Piran solution was added to the cells. Then, the cells were incubated for 30 min with 100 µl of RNase at 4 °C avoiding light for flow cytometry detection.

### Flow cytometry to detect apoptosis

An Annexin V-FITC Apoptosis Detection Kit (KeyGen Biotech, Nanjing, China, KGA108) was used to detect cell apoptosis. Cells were inoculated on a six-well plate and washed twice with pre-cooled PBS at 24 h after transfection. The cells were digested with trypsin without EDTA, centrifuged at 300×*g* for 5 min at 4 °C, washed once with pre-cooled PBS, and centrifuged at 4 °C at 300×*g* for 5 min. Then, 250 µl of binding buffer was added to the cells to resuspend them, and 100 µl of cells was added to a flow tube. A PI solution (5 µl) and Annexin V-Alexa Fluor (10 µl) were mixed and reacted with the cells at room temperature for 15 min, after which flow cytometry analysis was performed.

### Western blot analysis

The culture medium from the Petri dish containing the cells was discarded, and pre-cooled PBS was added to wash the cells three times for 5 min each. ESCA cells (ECA109 and TE-13) were resuspended in RIPA buffer and a protease inhibitor cocktail at a ratio of 100:1, and the ECA109 and TE-13 cells were lysed on ice for 30 min. The cells were scraped off of the dish and placed into a 1.5-ml EP tube. Then, the supernatant after centrifugation at 4 °C and 12,000×*g* for 15 min was obtained. Next, 5 × SDS buffer was added to the supernatant at a ratio of 4:1 according to the amount of supernatant, and the samples were placed in an incubator at 95 °C for 5 min. Finally, the protein concentration was determined with the BCA method. The volume of the solution containing 50 µg of protein was used as the sample volume. Samples were separated by SDS–PAGE at 70 V for 30 min and 110 V for 90 min. The proteins were transferred to a PVDF membrane by electroporation using an eBlot rapid protein transfer system. After transfer to the membrane, the membrane containing a band for the target protein was incubated with 1% skim milk (232,100) for 2 h. TBST was used to wash the membranes three times for 5 min each. The membranes were incubated with antibodies against E2F1 (Cell Signaling Technology, USA, 1:1000, 3742 s), Cyclind1 (Cell Signaling Technology, USA, 1:1000, 2978 T) and P21 (Cell Signaling Technology, USA, 1:1000, 2947 s) in a shaker at 4 °C overnight. After washing three times, the membranes were incubated with TBST containing goat anti-rabbit IgG and anti-horseradish peroxidase (HRP) while protected from light for 2 h. TBST was used to wash the membranes three times for 5 min each, and the membranes were finally scanned with an Odyssey fluorescence scanner.

### RIP

The Magna RIP RNA-Binding Protein Immunoprecipitation Kit (Millipore) was used to determine whether SBF2-AS1 and miR-362-3P/miR-338-3P can bind the AGO2 protein. The cells were washed twice with pre-cooled PBS for 5 min each, and the supernatant was discarded to collect the cells. The cells were lysed on ice with RIP lysis buffer for 5 min, and the cells were then frozen at − 80 °C. Fifty microlitres of magnetic beads was washed once with 500 µl of RIP wash buffer, placed on a magnetic stand, and the supernatant was discarded. Magnetic beads (50 µl) and antibodies (5 µg) were combined and incubated at room temperature for 30 min. The cell lysate was removed from − 80 °C and centrifuged at 14,000 rpm for 10 min at 4 °C. Then, 100 µl of cell lysate and RIP immunoprecipitation buffer containing magnetic beads were mixed and incubated overnight in a 4 °C incubator while shaking. The RNA from the magnetic bead-immunoprecipitation complexes was extracted by proteinase K restriction endonuclease digestion. The RNA was purified with phenol:chloroform:isoamyl alcohol (125:24:1). RNA was precipitated with salt solution I, precipitate enhancer and anhydrous ethanol, after which RT-PCR was carried out. Antibodies against AGO2 (Abcam) and IgG (Abcam) were used during the assay.

### RNA-pulldown assays

Pulldown assays were used to assess the binding of SBF2-AS1 with miR-362-3P and miR-338-3P. First, biotin-labelled miR-338-3P and miR-362-3P (RiboBio, Guangzhou, China) was designed. Magnetic beads were resuspended with lysis buffer (20 mM Tris (pH 7.5), 100 mM KCl, 5 m MgCl2, 0.3% NP-40, 50 U of RNaseOUT (Invitrogen, USA,) and incubated at 4 °C and 15 rpm for 2 h. The cells were cleaved by incubation with lysis buffer for 5 min on ice. The magnetic beads were mixed with the cell lysate and incubated at 15 rpm at 4 °C for 4 h. Then, TRIzol was added to extract the RNA, and RT-PCR was performed.

### Dual-luciferase reporter assays

A Dual-Luciferase Assay Kit (Promega, USA,) was used to verify the binding of miR-362-3P andmiR-338-3P with E2F1. First, the binding site of E2F1 for miR-362-3P and miR-338-3P was mutated, and the mutant and wild-type sequences were inserted into the pGLO fluorescent vector (Ruizhen, Nanjing, China). The culture medium of the cells was removed, and the cells were washed twice with pre-cooled PBS. The cells were lysed with 1 × PLB for 15 min, and 20 µl of cell lysate was added to a 96-well plate for immunofluorescence. Then, 100 µl of LAR II and StopGlo were added to measure fluorescence.

### Establishment of xenograft tumours in nude mice

A subcutaneous tumour-bearing model confirmed that SBF2-AS1 can promote the proliferation of oesophageal cancer in vivo. Seven-week-old male SPF-grade Balb/C nude mice were purchased from Yunqiao (Nanjing, China). The animal experiments described in the manuscript were approved by the Animal Research Ethics Committee of Bengbu Medical College. The study was carried out in compliance with the ARRIVE guidelines. ECA109 cells at logarithmic growth phase were collected and subcutaneously injected into nude mice at 5 × 10^5^ cells per mouse. Measure the size and volume of the mouse tumor every 3 days. When the mouse tumor grows to 100 mm^3^, randomly divide 10 mice into two groups. SBF2-AS1ASO and SBF2-AS1ASO-NC (RiboBio, Guangzhou, China) were given to mice in the SBF2-AS1ASO and SBF2-AS1ASO-NC groups at 1.5 kg/ml twice a week for three weeks. The body weight and tumor size and volume of the mice were measured every 3 days. Finally, the mice were anesthetized and euthanized. The tumors of the mice were removed and the pictures were taken.

### Immunohistochemistry

Paraffinized slices were dewaxed in water and incubated with 3% H_2_O_2_ at room temperature for 10 min. The slices were rinsed with distilled water and soaked in PBS twice for 5 min each. Then, 5% normal goat serum was closed and incubated at room temperature for 10 min. The serum was removed, and antibody was added and incubated with the cells at 4 °C overnight. The slices were washed with PBS three times, and an appropriate amount of horseradish peroxidase-labelled streptavidin was added and incubated with the slices at 37 °C for 30 min, followed by development with a chromogenic agent for 15 min. Finally, the sections were rinsed with water, re-dyed, dehydrated, cleared and sealed.

### Statistical analyses

SPSS 16.0 software was used for the statistical analyses in this study. This study used many statistical research methods. The Kaplan–Meier method was used to estimate survival probability, and one-way ANOVA or Student's t-test was used for intergroup comparisons. All data are shown as the average ± standard error. P < 0.05 was used to indicate statistical significance (Supplementary File S1).

## Supplementary Information

Supplementary Information

## Data Availability

All data generated or analysed during this study are included in this published article (and its Supplementary Information files).

## References

[1] Bray F, Ferlay J, Soerjomataram I, Siegel RL, Torre LA, Jemal A (2018). Global cancer statistics 2018: GLOBOCAN estimates of incidence and mortality world wide for 36 cancers in 185 countries. CA Cancer J. Clin..

[2] Reichenbach ZW, Murray MG, Saxena R (2019). Clinical and translational advances in esophage al squamous cell carcinoma. Adv. Cancer Res..

[3] Arnold M, Laversanne M, Brown LM, Devesa SS, Bray F (2017). Predicting the future burden of esophageal cancer by histological subtype: International trends in incidence up to 2030. Am. J. Gastroenterol..

[4] Rubinstein MR, Wang X, Liu W, Hao Y, Cai G, Han YW (2013). Fusobacterium nucleatum promotes colorectal carcinogenesis by modulating E-cadherin/β-catenin signaling via its FadA adhesin. Cell Host Microbe..

[5] Rustgi AK, El-Serag HB (2014). N. Engl. J. Med..

[6] Esteller M (2011). Non-coding RNAs in human disease. Nat. Rev. Genet..

[7] Wapinski O, Chang HY (2011). Long noncoding RNAs and human disease. Trends Cell Biol..

[8] Wang KC, Chang HY (2011). Molecular mechanisms of long noncoding RNAs. Mol. Cell..

[9] Dai JH, Huang WZ, Li C (2019). Silencing of long noncoding RNA SBF2-AS1 inhibits proliferation, migration and invasion and contributes to apoptosis in osteosarcoma cells by upregulating microRNA-30a to suppress FOXA1 expression. Cell Cycle.

[10] Chen R, Xia W, Wang S (2019). Long noncoding RNA SBF2-AS1 is critical for tumorigenesis of early-stage lung adenocarcinoma. Mol. Ther. Nucleic Acids..

[11] Yu H, Zheng J, Liu X (2017). Transcription factor NFAT5 promotes glioblastoma cell-driven angiogenesis via SBF2-AS1/miR-338-3p-mediated EGFL7 expression change. Front. Mol. Neurosci..

[12] Zhang Z, Yin J, Lu C, Wei Y, Zeng A, You Y (2019). Exosomal transfer of long non-coding RNA S BF2-AS1 enhances chemoresistance to temozolomide in glioblastoma. J. Exp. Clin. Cancer Res..

[13] Lv J, Qiu M, Xia W (2016). High expression of long non-coding RNA SBF2-AS1 promotes proliferation in non-small cell lung cancer. J. Exp. Clin. Cancer Res..

[14] Salmena L, Poliseno L, Tay Y, Kats L, Pandolfi PP (2011). A ceRNA hypothesis: The rosetta stone of a hidden RNA language?. Cell.

[15] Li Y, Liu JJ, Zhou JH, Chen R, Cen CQ (2020). LncRNA HULC induces the progression of osteosar coma by regulating the miR-372-3p/HMGB1 signalling axis. Mol. Med..

[16] Zhang J, Xu C, Gao Y (2020). A novel long non-coding RNA, MSTRG.51053.2 regulates cis platin resistance by sponging the miR-432-5p in non-small cell lung cancer cells. Front. Oncol..

[17] Dong X, Yang Z, Yang H, Li D, Qiu X (2020). Long non-coding RNA MIR4435-2H GPromotes colorectal cancer proliferation and metastasis through miR-206/YAP1 axis. Front. Oncol..

[18] Wang S, Qi Y, Gao X (2020). Hypoxia-induced lncRNA PDIA3P1 promotes mesenchymal transition via sponging of miR-124-3p in glioma. Cell Death Dis..

[19] Ferlay J, Soerjomataram I, Dikshit R (2015). Cancer incidence and mortality world wide: sources, methods and major patterns in GLOBOCAN 2012. Int. J. Cancer..

[20] Yang J, Lin J, Liu T (2014). Analysis of lncRNA expression profiles in non-small cell lung cancers (NSCLC) and their clinical subtypes. Lung Cancer..

[21] Harries LW (2012). Long non-coding RNAs and human disease. Biochem. Soc. Trans..

[22] Yang X, Tao H, Wang C, Chen W, Hua F, Qian H (2020). lncRNA-ATB promotes stemness maintenance in colorectal cancer by regulating transcriptional activity of the β-catenin pathway. Exp. Ther. Med..

[23] Wu Y, Hao N, Wang S (2020). Long noncoding RNA Lnc-TLN2-4:1 suppresses gastric cancer metastasis and is associated with patient survival. J. Oncol..

[24] Tang W, Yu X, Zeng R, Chen L (2020). LncRNA-ATB promotes cisplatin resistance in lung adenocarcinoma cells by targeting the miR-200a/β-catenin pathway. Cancer Manag. Res..

[25] Zhao J, Yang T, Li L (2020). LncRNA FOXP4-AS1 is involved in cervical cancer progression via regulating miR-136-5p/CBX4 axis. Oncol. Targets Ther..

[26] Xiu D, Liu L, Cheng M, Sun X, Ma X (2020). Knockdown of lncRNA TUG1 enhances radio sensitivity of prostate cancer via the TUG1/miR-139-5p/SMC1A axis. Oncol. Targets Ther..

[27] Xia W, Liu Y, Cheng T, Xu T, Dong M, Hu X (2020). Down-regulated lncRNA SBF2-AS1 inhibits tumorigenesis and progression of breast cancer by sponging microRNA-143 and repressing RR S1. J. Exp. Clin. Cancer Res..

[28] Yu ZW, Wang GB, Zhang CL (2020). LncRNA SBF2-AS1 affects the radio sensitivity of non-small cell lung cancer via modulating microRNA-302a/MBNL3 axis. Cell Cycle.

[29] Liang C, Yue C, Liang C (2019). The long non-coding RNA SBF2-AS1 exerts oncogenic functions in gastric cancer by targeting the miR-302b-3p/E2F transcription factor 3 axis. Oncol. Targets Ther..

[30] Shen WJ, Zhang F, Zhao X, Xu J (2016). LncRNAs and esophageal squamous cell carcinoma: Implications for pathogenesis and drug development. J. Cancer..

[31] He M, Feng L, Qi L, Rao M, Zhu Y (2020). Long noncoding RNASBF2-AS1 promotes gastric cancer progression via regulating miR-545/EMS1 axis. Biomed. Res. Int..

[32] Lin RJ, Xiao DW, Liao LD, Chen T, Xie ZF, Huang WZ, Wang WS, Jiang TF, Wu BL, Li EM (2012). MiR-142-3p as a potential prognostic biomarker for esophageal squamous cell carcinoma. J. Surg. Oncol..

[33] Zhang C, Li H, Wang J, Zhang J, Hou X (2019). MicroRNA-338-3p suppresses cell proliferation, migration and invasion in human malignant melanoma by targeting MACC1. Exp. Ther. Med..

[34] Assiri AA, Mourad N, Shao M (2019). MicroRNA 362–3p reduces hERG-related current and inhibits breast cancer cells proliferation. Cancer Genomics Proteomics..

[35] Wang D, Wang H, Li Y, Li Q (2018). MiR-362-3p functions as a tumor suppressor through targeting MCM5 in cervical adenocarcinoma. Biosci. Rep..

[36] Chan J, Tay Y (2018). Noncoding RNA: RNA regulatory networks in cancer. Int. J. Mol. Sci..

[37] Hollern DP, Swiatnicki MR, Rennhack JP (2019). E2F1 drives breast cancer metastasis by reg ulating the target gene FGF13 and altering cell migration. Sci. Rep..

[38] Xia L, Nie D, Wang G, Sun C, Chen G (2019). FER1L4/miR-372/E2F1 works as a ceRNA system to regulate the proliferation and cell cycle of glioma cells. J. Cell Mol. Med..

[39] Chen B, Zhao Q, Guan L (2018). Long non-coding RNA NNT-AS1 sponges miR-424/E2F1 to promote the tumorigenesis and cell cycle progression of gastric cancer. J. Cell Mol. Med..

