# Supplementary material for: The molecular mechanisms of the long noncoding RNA SBF2-AS1 in regulating the proliferation of oesophageal squamous cell carcinoma
Source: Sci Rep. 2021 Jan 12;11:805. doi: 10.1038/s41598-020-80817-w (PMC7804443; doi:10.1038/s41598-020-80817-w)
Supplement: Supplementary file 1 — Supplementary Information [file 41598_2020_80817_MOESM1_ESM.pdf]

# The molecular mechanisms of the long noncoding RNA SBF2-AS1 in regulating the proliferation of oesophageal squamous cell carcinoma

**Wenjuan Zha[1+], Xiaomin Li[1+], Xiaowei Tie[1+], Yao Xing[1], Hao Li[2], Fei Gao[3], Ting Ye[2], Wangqi Du[2], Rui Chen[4*], Yangchen Liu[3*]**

[1]Department of Radiotherapy, Taixing People's Hospital Affiliated with Bengbu Medical College, Bengbu China

[2]Department of Clinical Laboratory, Taixing People's Hospital, Taixing, China

[3]Department of Radiotherapy, Taixing People's Hospital, Taixing, China

[4]Department of Taixing People's Hospital Affiliated with Yangzhou University, Yangzhou, China

***Correspondence: Yangchen Liu and Rui Chen**

**Yangchen Liu:** Department of Radiotherapy, Taixing People's Hospital, Taixing (225400), China. Tel: +86-13961001669, E-mail: liuyctx@163.com

**Rui Chen:** Department of Taixing People's Hospital Affiliated with Yangzhou University, Yangzhou (225000), China. Tel: +86-0278669870, E-mail: 406600245@qq.com

**[+]These authors contributed equally to this work**

## Supplementary information1

**TE-13:E2F1-SI-NC-EV-OE(70kDa)**

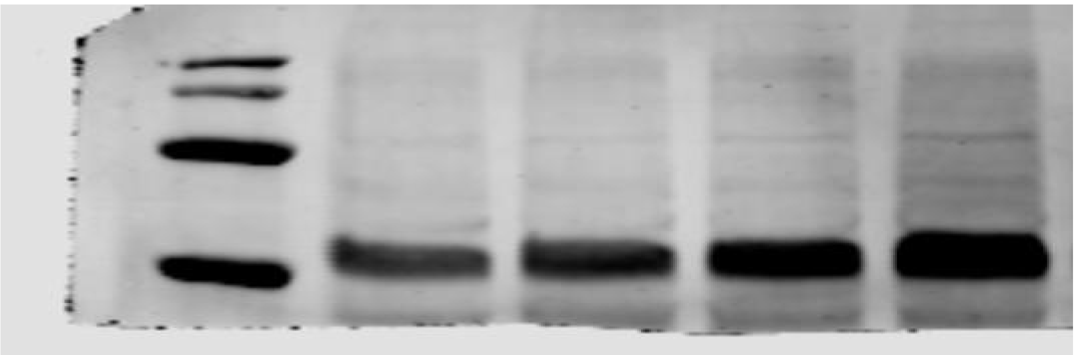

**ECA109:E2F1-SI-NC-EV-OE**

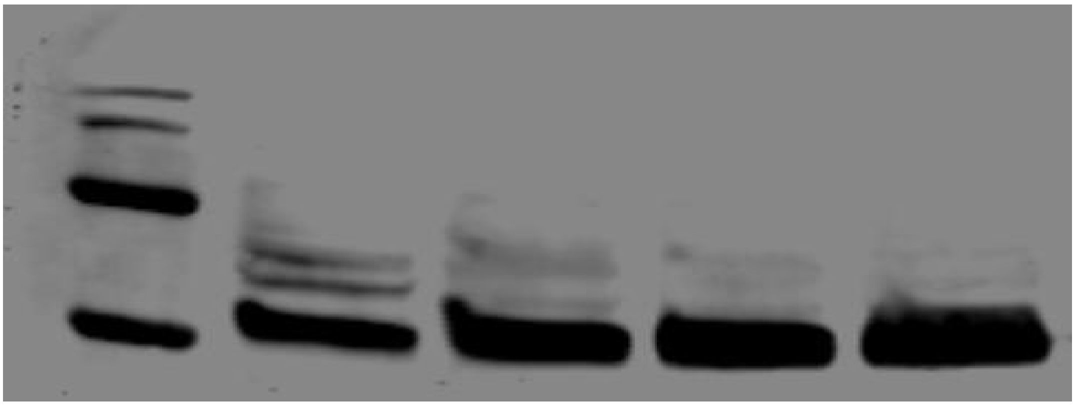

**TE-13:Cyclind1-SI-NC-EV-OE(36kDa)**

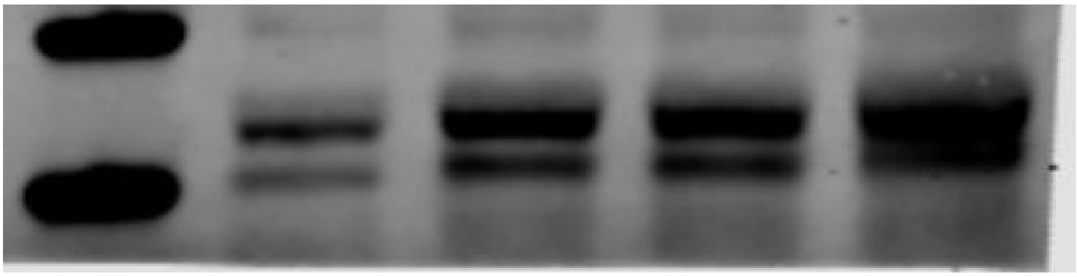

**ECA109:Cyclind1-SI-NC-EV-OE**

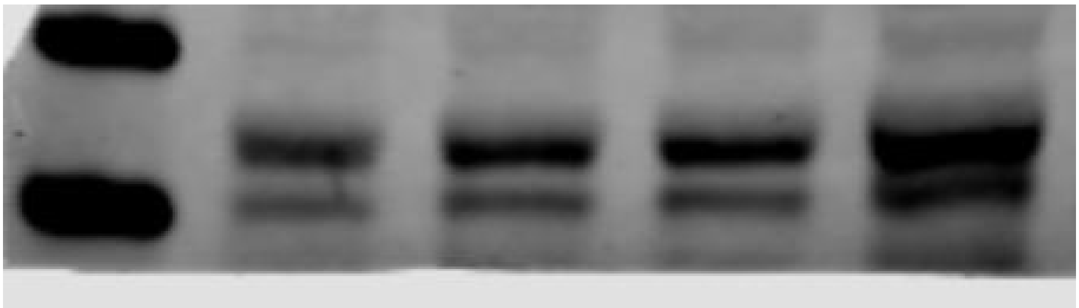

TE-13:P21-SI-NC-EV-OE(kDa)　　ECA109:P21-SI-NC-EV-OE

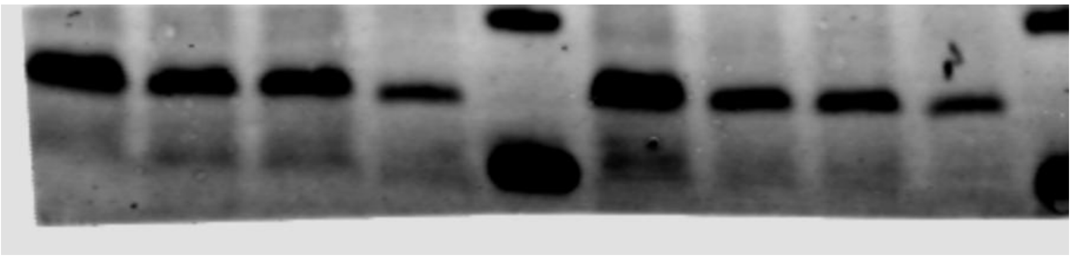

TE-13:ACTIN-SI-NC-EV-OE(43kDa)

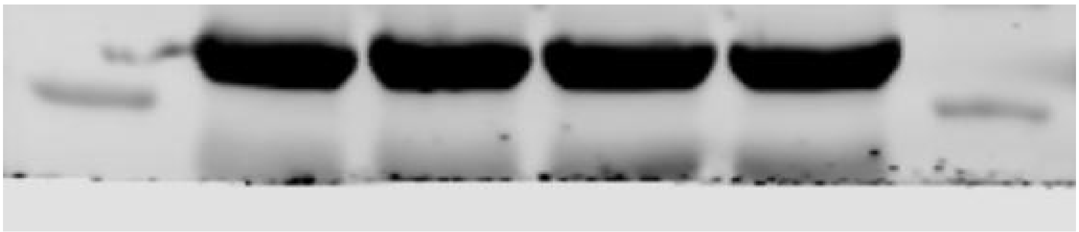

ECA109:ACTIN-SI-NC-EV-OE

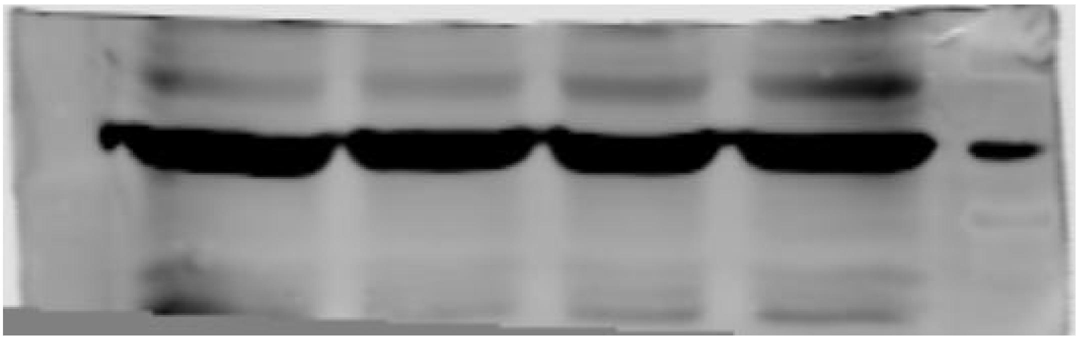

TE-13:E2F1-EV-OE-OE+miR-362-3P-OE+miR-338-3P-OE+mimic nc

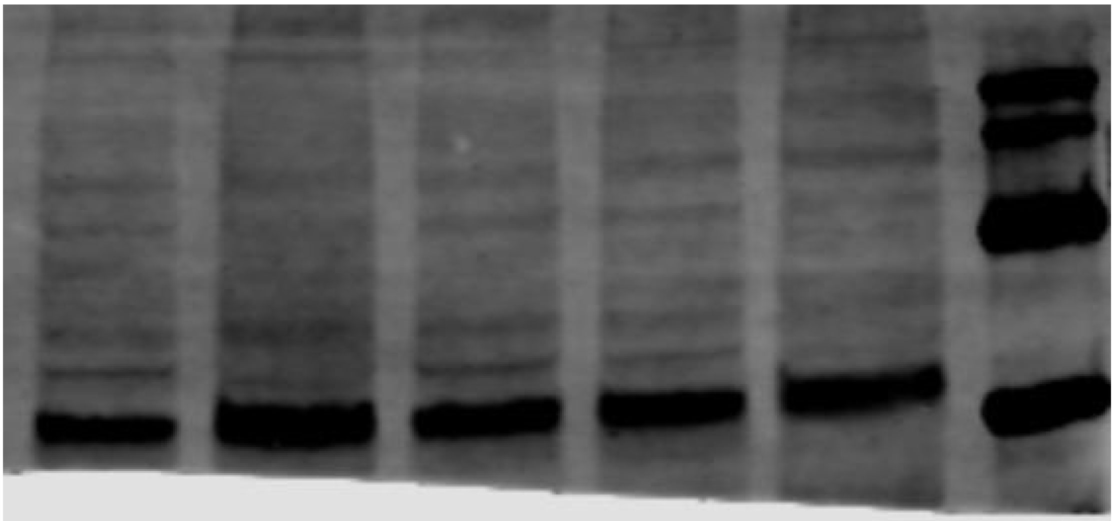

ECA109-E2F1-EV-OE-OE+miR-362-3P-OE+miR-338-3P-OE+mimic nc

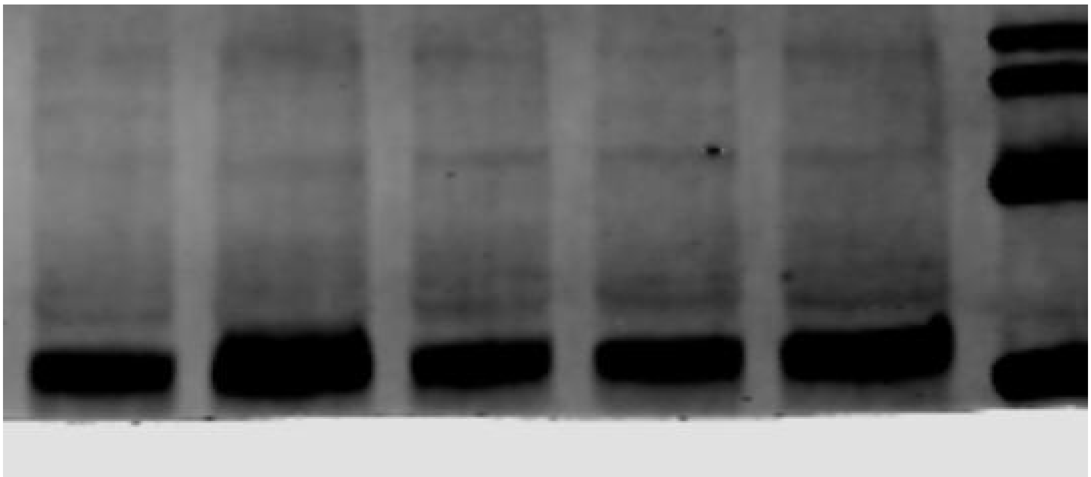

TE-13:Cyclind1-EV-OE-OE+miR-362-3P-OE+miR-338-3P-OE+mimic nc

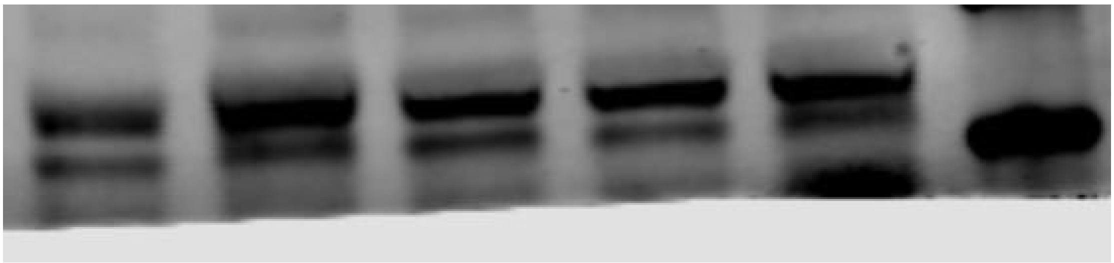

ECA109:Cyclind1-EV-OE-OE+miR-362-3P-OE+miR-338-3P-OE+mimic nc

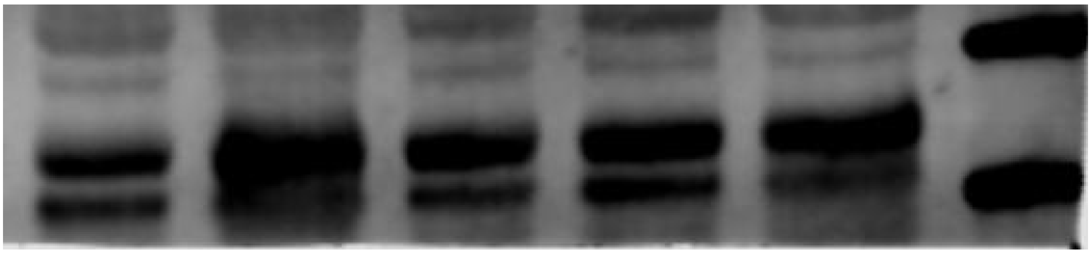

TE-13:P21-EV-OE-OE+miR-362-3P-OE+miR-338-3P-OE+mimic nc

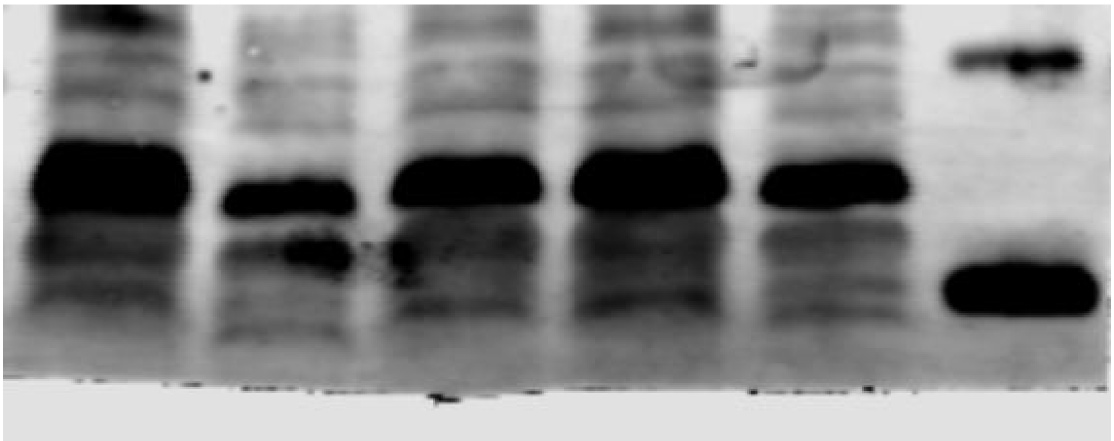

ECA109:P21-EV-OE-OE+miR-362-3P-OE+miR-338-3P-OE+mimic nc

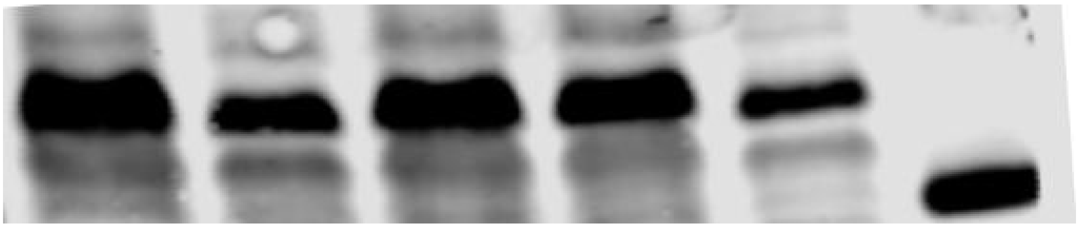

TE-13:ACTIN-EV-OE-OE+miR-362-3P-OE+miR-338-3P-OE+mimic nc

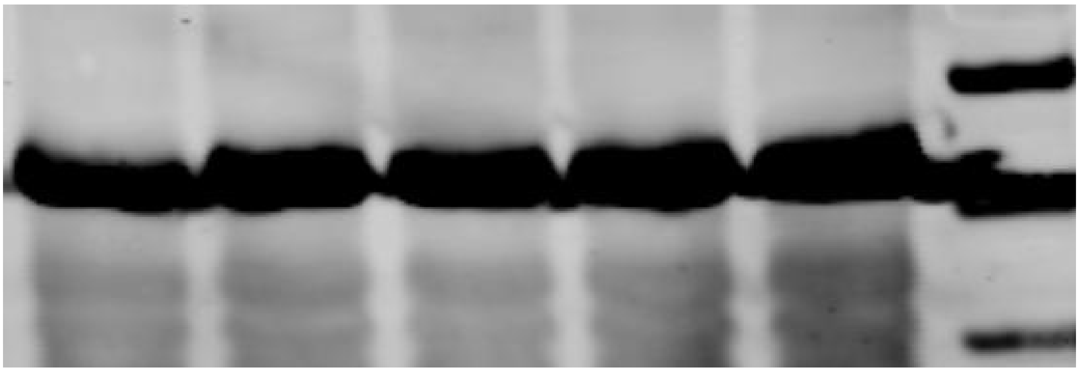

ECA109:ACTIN-EV-OE-OE+miR-362-3P-OE+miR-338-3P-OE+mimic nc

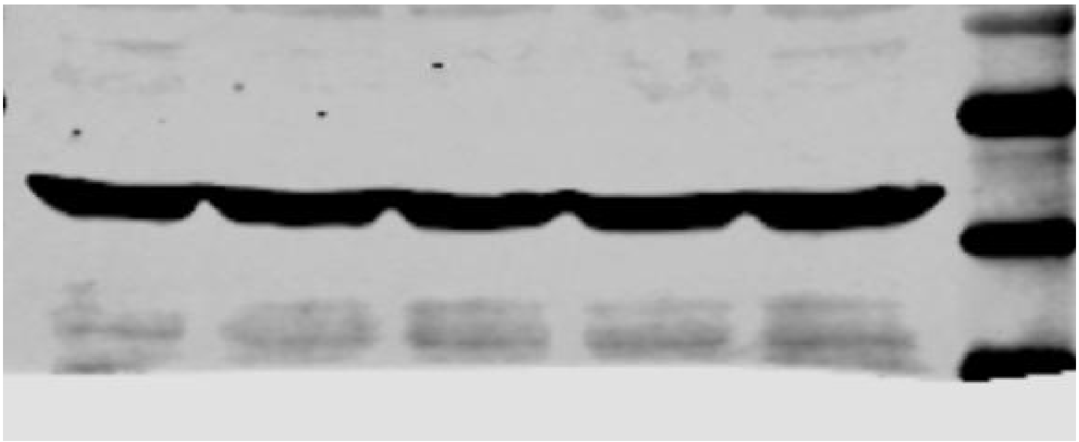

## Supplementary information2

## Constructed plasmids vectors used in this study

E2F1-WT, wild type E2F1 3'UTR

CAGGGCTTGGAGGGACCAGGGTTTCCAGAGATGCTCACCTTGTCTCTGCA GCCCTGGAGCCCCC

TGTCCCTGGCCGTCCTCCCAGCCTGTTTGGAAACATT TAATTTATACCCCTCTCCTCTGTCTCCAGA

AGCTTCTAGCTCTGGGGTCTGG CTACCGCTAGGAGGCTGAGCAAGCCAGGAAGGGAAGGAGTCT

GTGTGGTG TGTATGTGCATGCAGCCTACACCCACACGTGTGTACCGGGGGTGAATGTGT GTGAG

CATGTGTGTGTGCATGTACCGGGGAATGAAGGTGAACATACACCTC TGTGTGTGCACTGCAGACA

CGCCCCAGTGTGTCCACATGTGTGTGCATGAG TCCATGTGTGCGCGTGGGGGGGCTCTAACTGC

ACTTTCGGCCCTTTTGCTC TGGGGGTCCCACAAGGCCCAGGGCAGTGCCTGCTCCCAGAATCTG

GTGCT CTGACCAGGCCAGGTGGGGAGGCTTTGGCTGGCTGGGCGTGTAGGACGGT GAGAGCA

CTTCTGTCTTAAAGGTTTTTTCTGATTGAAGCTTTAATGGAGCGT TATTTATTTATCGAGGCCTCTTTG

GTGAGCCTGGGGAATCAGCAAAGGGGA GGAGGGGTGTGGGGTTGATACCCCAACTCCCTCTACC

CTTGAGCAAGGGC AGGGGTCCCTGAGCTGTTCTTCTGCCCCATACTGAAGGAACTGAGGCCTGG

GTGATTTATTTATTGGGAAAGTGAGGGAGGGAGACAGACTGACTGACAGC CATGGGTGGTCAGAT

GGTGGGGTGGGCCCTCTCCAGGGGGCCAGTTCAGG GCCCCAGCTGCCCCCCAGGATGGATATG

AGATGGGAGAGGTGAGTGGGGG ACCTTCACTGATGTGGGCAGGAGGGGTGGTGAAGGCCTCCC

CCAGCCCAG ACCCTGTGGTCCCTCCTGCAGTGTCTGAAGCGCCTGCCTCCCCACTGCTCT GCC

CCACCCTCCAATCTGCACTTTGATTTGCTTCCTAACAGCTCTGTTCCCT CCTGCTTTGGTTTTAATAA

ATATTTTGATGACGTT

E2F1-MUT1, binding site of miR-338-3p was deletion-mutated

CAGGGCTTGGAGGGACCAGGGTTTCCAGAGATGCTCACCTTGTCTCTGCA GCCCTGGAGCCCCC

TGTCCCTGGCCGTCCTCCCAGCCTGTTTGGAAACATT TAATTTATACCCCTCTCCTCTGTCTCCAGA

AGCTTCTAGCTCTGGGGTCTGG CTACCGCTAGGAGGCTGAGCAAGCCAGGAAGGGAAGGAGTCT

GTGTGGTG TGTATGTGCATGCAGCCTACACCCACACGTGTGTACCGGGGGTGAATGTGT GTGAG

CATGTGTGTGTGCATGTACCGGGGAATGAAGGTGAACATACACCTC TGTGTGTGCACTGCAGACA

CGCCCCAGTGTGTCCACATGTGTGTGCATGAG TCCATGTGTGCGCGTGGGGGGGCTCTAACTGC

ACTTTCGGCCCTTTTGCTC TGGGGGTCCCACAAGGCCCAGGGCAGTGCCTGCTCCCAGAATCTG

GTGCT CTGACCAGGCCAGGTGGGGAGGCTTTGGCTGGCTGGGCGTGTAGGACGGT GAGAGCA

CTTCTGTCTTAAAGGTTTTTTCTGATTGAAGCTTTAATGGAGCGT TATTTATTTATCGAGGCCTCTTTG

GTGAGCCTGGGGAATCAGCAAAGGGGA GGAGGGGTGTGGGGTTGATACCCCAACTCCCTCTACC

CTTGAGCAAGGGCAGGGGTCCCTGAGCTGTTCTTCTGCCCCATACACTGAGGCCTGGGTGATTT A

TTTATTGGGAAAGTGAGGGAGGGAGACAGACTGACTGACAGCCATGGGT GGTCAGATGGTGGGG

TGGGCCCTCTCCAGGGGGCCAGTTCAGGGCCCCAG CTGCCCCCCAGGATGGATATGAGATGGGA

GAGGTGAGTGGGGGACCTTCA CTGATGTGGGCAGGAGGGGTGGTGAAGGCCTCCCCCAGCCCA

GACCCTGT GGTCCCTCCTGCAGTGTCTGAAGCGCCTGCCTCCCCACTGCTCTGCCCCAC CCTC

CAATCTGCACTTTGATTTGCTTCCTAACAGCTCTGTTCCCTCCTGCTT TGGTTTTAATAAATATTTTGA

TGACGTT

E2F1-MUT2, binding sites of miR-362-3p were deletion-mutated

CAGGGCTTGGAGGGACCAGGGTTTCCAGAGATGCTCACCTTGTCTCTGCA GCCCTGGAGCCCCC

TGTCCCTGGCCGTCCTCCCAGCCTGTTTGGAAACATT TAATTTATACCCCTCTCCTCTGTCTCCAGA

AGCTTCTAGCTCTGGGGTCTGG CTACCGCTAGGAGGCTGAGCAAGCCAGGAAGGGAAGGAGTCT

GTGTGTGT GCATGCAGCCTACACCCACACCCGGGGGTGAATGTGTGTGAGCATGTGTGT GTGCA

TGTACCGGGGAATGAAGGTGAACATACACCTCTGTGTGTGCACTGC AGACACGCCCCAGTGTGTC

CACATGTGTGTGCATGAGTCCATGTGTGCGCG TGGGGGGGCTCTAACTGCACTTTCGGCCCTTTT

GCTCTGGGGGTCCCACAA GGCCCAGGGCAGTGCCTGCTCCCAGAATCTGGTGCTCTGACCAGG

CCAGG TGGGGAGGCTTTGGCTGGCTGGGCGTGTAGGACGGTGAGAGCACTTCTGT CTTAAAGG

TTTTTTCTGATTGAAGCTTTAATGGAGCGTTATTTATTTATCGAG GCCTCTTTGGTGAGCCTGGGGAA

TCAGCAAAGGGGAGGAGGGGTGTGGG GTTGATACCCCAACTCCCTCTACCCTTGAGCAAGGGCA

GGGGTCCCTGAGC TGTTCTTCTGCCCCATACTGAAGGAACTGAGGCCTGGGTGATTTATTTATTG

GGAAAGTGAGGGAGGGAGACAGACTGACTGACAGCCATGGGTGGTCAGA TGGTGGGGTGGGCC

CTCTCCAGGGGGCCAGTTCAGGGCCCCAGCTGCCCC CCAGGATGGATATGAGATGGGAGAGGTG

AGTGGGGGACCTTCACTGATGT GGGCAGGAGGGGTGGTGAAGGCCTCCCCCAGCCCAGACCCT

GTGGTCCCT CCTGCAGTGTCTGAAGCGCCTGCCTCCCCACTGCTCTGCCCCACCCTCCAA TCTG

CACTTTGATTTGCTTCCTAACAGCTCTGTTCCCTCCTGCTTTGGTTTTA ATAAATATTTTGATGACGTT

## Supplementary information3

## Primers and siRNA sequences

| Supplementary Table1. Primers and siRNA sequences | | |
|---|---|---|
| | | |
| siRNA sequcnee | Sense 5'-3' | Antisense 5'-3' |
| siRNA sequcnee | CAGAAGGAGUCUACUGCUAAG | UAGCAGUAGACUCCUUCUGGG |
| SBF2-AS1(1021) | GCAAGCCUGCAUGGUACAUTT | AUGUACCAUGCAGGCUUGCTT |
| PCR primers | | |
| Gene | Forward 5'-3' | Reserve 5'-3' |
| SBF2AS1 | CACGACCCAGAAGGAGTCTAC | CCCGGTACCTTCCTGTCATA |
| B-Actin | GAAATCGTGCGTGACATTAA | AAGGAAGGCTGGAAGAGTG |
| | | |

<table>
<tr><td colspan="2">Supplementary Table2.the binding sites of E2F1/SBF2-AS1 and miR-3</td></tr>
<tr><td>1</td><td></td></tr>
<tr><td></td><td>miR-338-3P 3’-gtTGTTTTAGT-GACTACGACCt-5’</td></tr>
<tr><td></td><td>:::|| || | ||||||||</td></tr>
<tr><td></td><td>SBF2-AS1 5’-tgGTGAACACATCAGATGCTGGg-3’</td></tr>
<tr><td>2</td><td></td></tr>
<tr><td></td><td>miR-362-3P 3’-acTTAGGAAC--TTATCCACACAa-5’</td></tr>
<tr><td></td><td>:|| || | |: |||||||</td></tr>
<tr><td></td><td>SBF2-AS1 5’-caGATGCTGGGCAGGTGGTGTGTt-3’</td></tr>
<tr><td>3</td><td></td></tr>
<tr><td></td><td>miR-338-3P 3’ guugUUUUAGUGACUACGACCu 5’</td></tr>
<tr><td></td><td>:|:: || | ||||||</td></tr>
<tr><td></td><td>E2F1 5’ gggaGAGGCCAAGGUUGCUGGg 3’</td></tr>
<tr><td>4</td><td></td></tr>
<tr><td></td><td>miR-362-3P 3’ acUUAGGAACUUAUCCACACAa 5’</td></tr>
<tr><td></td><td>|:|| || :| |||||||</td></tr>
<tr><td></td><td>E2F1 5’ ggAGUC--UGUGU-GGUGUGUa 3’</td></tr>
<tr><td></td><td></td></tr>
</table>

**Supplementary information4**

# Related terms

**1.RTCA:** Real-time unlabeled cell analysis technique , It is mainly used to measure cell proliferation

**2. EDU:** 5-Ethynyl-2'-deoxyuridine, Detection of cell proliferation ability

**3. ASO:** Antisense oligonucleotide (ASO) is an effect┐ive and feasible way to target a gene of interest selectively. ASOs have proven to cleave target RNA in both the cyto┐plasm and nucleus depending on RNase H1

**4. AGO2:** AGO2 is the core element of RNA-induced silencing complex, which mainly promotes the degradation of target miRNA or inhibits its

protein translation in miRNA/siRNA pathway. Whether lncRNA can bind to AGO2 protein is usually regarded as an important marker to play the role of ceRNA.

**5.ceRNA:** ceRNA is a kind of endogenous RNA that can bind miRNA through MRE site, including mRNA, non-coding RNA, pseudogene and circular RNA.
